# Epigenetic hereditary transcription profiles III, evidence for an epigenetic network resulting in gender, tissue and age-specific variation in overall transcription

**DOI:** 10.1186/1745-6150-4-37

**Published:** 2009-10-01

**Authors:** Johannes WIM Simons

**Affiliations:** 1 Department of Toxicogenetics, MGC, Leiden University Medical Center, PO Box 9600, 2300 RC Leiden, The Netherlands

## Abstract

**Background:**

We have previously shown that deviations from the average transcription profile of a group of functionally related genes are not only heritable, but also demonstrate specific patterns associated with age, gender and differentiation, thereby implicating genome-wide nuclear programming as the cause. To determine whether these results could be reproduced, a different micro-array database (obtained from two types of muscle tissue, derived from 81 human donors aged between 16 to 89 years) was studied.

**Results:**

This new database also revealed the existence of age, gender and tissue-specific features in a small group of functionally related genes. In order to further analyze this phenomenon, a method was developed for quantifying the contribution of different factors to the variability in gene expression, and for generating a database limited to residual values reflecting constitutional differences between individuals. These constitutional differences, presumably epigenetic in origin, contribute to about 50% of the observed residual variance which is connected with a network of interrelated changes in gene expression with some genes displaying a decrease or increase in residual variation with age.

**Conclusion:**

Epigenetic variation in gene expression without a clear concomitant relation to gene function appears to be a widespread phenomenon. This variation is connected with interactions between genes, is gender and tissue specific and is related to cellular aging.

This finding, together with the method developed for analysis, might contribute to the elucidation of the role of nuclear programming in differentiation, aging and carcinogenesis

**Reviewers:**

This article was reviewed by Thiago M. Venancio (nominated by Aravind Iyer), Hua Li (nominated by Arcady Mushegian) and Arcady Mushegian and J.P.de Magelhaes (nominated by G. Church).

## Background

The phenomenon of nuclear programming; i.e. the persistent epigenetic system that controls and maintains the differentiated state; has been clearly demonstrated by the ability to clone animals via transfer of somatic cell nuclei into enucleated egg cells. Elucidating the process which underlies this important epigenetic mechanism is crucial for reprogramming somatic cells into pluripotent cells for biomedical and agricultural applications and invaluable in biological research on development, aging and disease, including cancer [1].

In order to understand the mechanism of epigenetic nuclear programming it is generally supposed that there exists a genome-wide complex network of higher-order gene regulation that controls global gene expression and, thereby, the cell's identity. At present, the methodology being used to help reveal this network of interacting genes involves studying the processes assumed to be of importance (e.g. nuclear architecture, chromatin structure, regulatory elements, DNA methylation, histone modification, genomic imprinting, etc.) and their connections with genome-wide transcriptome and proteome analysis.

This approach however, is only in its infancy, and there exists a need for additional data analysis strategies in order to obtain an integrated picture of nuclear programming.

The recent, serendipitous discovery of "epigenetic hereditable transcription profiles" suggests such an additional strategy [2,3] and will in the sequel be referred to as EPIGENE (Epigenetic Programs in GENe Expression). The EPIGENE method does not try disentangle a large complex network of interacting genes in gene regulation, instead, it studies the variation in expression in a small group of functionally related genes.

To date, only the genes coding for a relatively simple cell organelle, the proteasome, have been studied. Surprisingly, a wide variation in the expression profiles of the genes between individual libraries was observed. This variation appears to lack a corresponding biological role, since the structure of the proteasome is, in all probability, the same in all individuals and tissues.

The main observations obtained using EPIGENE were that:-

- the deviations from the mean transcription profile for this set of genes in a database display a network of interrelated variations in expression of the genes

- such a specific network is hereditary since it can be transmitted to daughter cells

- different tissues demonstrate specific profiles

- gender-specific deviations from the mean transcription profile are present

- aging is correlated with specific changes in the transcription profiles

- tissue samples demonstrating disturbed profiles are observed.

This data suggests that epigenetic programming is essential in the expression of the majority of the genes and can therefore be studied in any subset of functionally related genes. Furthermore it indicates that the specificity in these expression profiles occurs autonomously rather than by ongoing regulatory signals.

Confirmation of these observations will validate EPIGENE as a useful strategy for studying several aspects of nuclear programming. In all probability, cell lineage trees could be identified by comparisons of transcription profiles. Such lineage trees might, for example, monitor foetal development or stages in carcinogenesis. The method might also prove useful in monitoring the reprogramming of somatic cells into pluripotent descendants and in identification of epigenetic abnormality.

The observations outlined above were obtained using a database derived from a large number of different tissues originating from different donors. The present investigation studies EPIGENE in a database derived only from muscle with the specific aims of:

- validating and expanding results obtained from previous studies

- improving EPIGENE analysis

- identifying sources of variation in expression profiles and quantifying their contribution to the total variation

For the present study, the transcription profile of genes that code for the 26S proteasome [4] was arbitrarily chosen as model system. Since a cellular organelle has a well-defined structure the genes are functionally related and it can be assumed that their expression is interconnected in such a way as to provide the correct amounts of the various components of the organelle. The characteristics of the genes and their location {as derived from Genatlas [5] } are given in Table 1.

**Table 1:** Characteristics of the genes and their location (as derived from Genatlas [5]).

**Gene**	**HGNC id**	**Chromosome Location**	**DNA (kb)**	**Number of Transcripts**	**mRNA**	**Number of Exons**	**Number of Amino Acids**	**Structural part of Proteasome**
								

PSMA1	9530	11p15,1	15,53	2	1453	11	269	ringA

					1210	10	263	ringA

PSMA2	9531	7p13	14,75	1	885	8	233	ringA

PSMA3	9532	14q23	27,04	2?	927	11	254	ringA

					906	11	248	ringA

PSMA4	9533	15q24,1	8,7	1	1172	9	261	ringA

PSMA5	9534	1p13	24,57	1	974	9	241	ringA

PSMA6	9535	14q13,2	25,13	1	1018	7	246	ringA

PSMA7	9536	20q133	6,59	2?	984	7	248	ringA

					1069	7		ringA

PSMB1	9537	6q27	18,12	1	882	6	241	ringB

PSMB2	9539	1p34	40,43	1	845	6	201	ringB

PSMB3	9540	17q12	11,42	1	753	6	205	ringB

PSMB4	9541	1q21	2,4	1	925	7	264	ringB

PSMB5	9542	14q11,2	8,79	1	1049	3	208	ringB

PSMB6	9543	17p13	2,31	1	824	6	239	ringB

PSMB7	9544	9q33	61,97	1	983	8	277	ringB

PSMC1	9547	19p13,3	16,07	1	1586	11	440	base

PSMC2	9548	7q22,1	20,49	1	1526	12	433	base

PSMC3	9549	11p,13	7,67	1	1545	12	439	base

PSMC4	9551	19q13,11	19,25	1	1433	11	418	base

PSMC5	9552	17q24	4,53	1	1319	12	406	base

PSMC6	9553	14q22,1	20,79	1	1579	14	389	base

PSMD1	9554	2q37,1	115,82	1	3227	25	953	lid

PSMD2	9559	3q27,3	9,79	1	2922	21	908	lid

PSMD3	9560	17q21,1	17,2	1	2147	12	534	lid

PSMD4	9561	1q21,2	12,74	2	1508	9	268	base

					1313	10	377	base

PSMD5	9563	9q33,3	26,8	1	3384	10	504	base

PSMD6	9564	3p14,1	12,9	1		8	389	lid

PSMD7	9565	16q23	9,5	1	1655	7	324	lid

PSMD8	9566	19q13,13	8,66	1	1301	7	257	lid

PSMD9	9567	12q24,31	29,1	2	2332	6	223	lid?

							209	lid

PSMD10	9555	Xq22,3	7,4	2	1467	5	151	lid

					1546	5	226	lid

PSMD11	9556	17q12	38	1	1580	14	422	lid

PSMD12	9557	17q24,3	28,7	2	3592	12	456	lid

					3532	11	436	lid

PSMD13	9558	11p15,5	15,96	2	1585	13	377	lid

					1670	12	351	lid

PSMD14	16889	2q24,3	103,7	1	1132	12	310	lid

The analysis proceeds as follows:

- comparison of the two subgroups of the database: abdominal muscle versus skeletal muscles of the extremities

- quantification of variation in expression and identification of contributing factors

- removal of the influence of contributing factors and analysis of the residual, presumably epigenetic, variation.

## Results and Discussion

### Comparison of the two muscle types

The complete gene expression database for 62 probes in 81 libraries (Database A) is given in Additional File 1. This database was derived from two different muscle sources i.e. rectus abdominis for M libraries and leg and arm muscles for V libraries. It was therefore necessary to first establish whether the two sources could be combined in the analysis. Comparison of the average expressions of each probe for the M and V libraries using the Wilcoxon test showed that for most probes, M libraries had higher mean expressions (Mann-Whitney, P < 0,0001).

Further an ANOVA with the 19 V libraries and 19 randomly chosen M libraries not only showed this difference in expression between M and V libraries (P = 4,27 × 10^-32^) but also demonstrated that the pattern of expressions of the probes are different for M and V libraries (P = 2,06 × 10^-12^). Consequently, since the libraries do differ substantially they must be analyzed separately as two distinct tissues.

To assess whether a gender related variation in transcript abundances exists and also males and females have to be analysed separately an ANCOVA was calculated for M libraries. An indication for interaction between age and gender was observed (P = 0,064). Although in the V libraries no significant interaction between gender and age was found (P = 0,236) a significant decrease with age was observed for the proteasome expression level in males (P = 0,0008) but not in females (P = 0,612).

Because of these differences, the database was also subdivided for gender resulting in four groups (M females, M males, V females and V males) that were analysed separately.

### Assessment of heterogeneity caused by proteasome expression level

The term "proteasome expression level" refers to the sum of all probe expression levels for a given library. The expression level showed a 2,6-fold and 2,5-fold variation in the M and V libraries respectively. A reasonable assumption as to the cause of this variation is that the proteasome expression level is related to the number of proteasomes in the cell. To remove this source of variation from the database, regression analysis was used to establish the relationship between the proteasome expression level and the expression of individual probes. These regressions were calculated for each of the four groups separately. To remove the contribution of proteasome expression level to the variability in expression, the residuals of these regression lines were added or subtracted from the mean expression of the probes. This data, i.e. without the influence of proteasome expression level, are presented as Database B (Additional file 2).

### Assessment of the contribution of diverse factors to the variability in expression

The expression levels of the individual probes differ widely, e.g. in M females the mean expression of PSMB1c is 35 whilst that of PSMA7a is 4787. Consequently, calculated variances are substantially influenced by the variability in expression of the individual probes. To eliminate this source of variability, the data for each of the four groups in Databases A and B (see Additional files 1 &2) were transformed in such a way that the mean expression of each probe became 1000. This procedure removed about 90% to 95% of the variability in the data. The transformed data are presented as Databases AA & BB in Additional files 1 &2. When the resulting variance in Database AA is set at 100% the contribution of proteasome expression level ranges from 38,7% for M males to 54,2% for V males (Table 2A). This means that 54-60% of the variability remains unaccounted for. One contributing factor must be experimental error (noise), but since the database does not include replicates this source of variation cannot be directly determined. However, an approximation of the experimental error can be obtained by comparing the expressions of two libraries that show a strong correlation.

**Table 2:** Factors contributing to the variability in expression of the proteasome genes.

**A. Database**		**Contents**	**Variance**	**df**	**percentage of variance**
Database AA		raw date transformed to a mean of 1000 per probe			
	M females		94652,5	1549	100,0
	M males		81873,5	2293	100,0
	V females		110677,7	681	100,0
	V males		79961,4	495	100,0
Database BB		additionally:proteasome expression level removed			
	M females		50437,8	1549	53,3
	M males		50182,3	2293	61,3
	V females		58000,6	681	52,4
	V males		36647,4	495	45,8
					
Experimental error	M females in AA	(approximation by use of 2 libraries, see text)	1302,2	61	1,4
					
**B.**					
Database BB	variable	F test			probability
	Gender	M males versus M females			0,9107
		V males versus V females			2,1693E-06
					
	Tissue	M males versus V males			0,0179
		M females versus V females			0,0002
					
	Race	M libraries non-whites	45008,6	1053	
		M libraries whites	1058207,3	1921	0,0135

A. After transformation of the databases to a mean expression level of 1000 per probe, the proteasome expression level appears to contribute about 50% to the variance while the experimental error contributes around 1,4%. The residual variation in expression, presumably related to epigenetic factors, is also around 50%.

B. Factors contributing to the residual variation in Database BB are tissue type, gender and race.

Within the group of M females the mean and standard deviation of the correlation coefficient is -0,035 and 0,477, therefore some libraries will be rather similar. The libraries M19 and M25 have 0,985 as correlation coefficient and have a highly significant regression (P = 2,31 × 10^-46^, after Bonferroni correction P = 7,49 × 10^-43^). This means that these two libraries are very much alike in expression and could approach the similarity of repeats. The variance for these two libraries, as determined from the variances of each individual probe is 1302 with 61 degrees of freedom. This indicates that the experimental error is only 1,4% (table 2A) and thus that over 50% of the variation in expression is due to other presumably epigenetic factors such as different tissue types (M and V libraries) and to constitutive differences between individuals (e.g. gender, age and genetic background.

This interpretation is further supported by the comparison of the variances of the four subgroups since significant differences were found for tissue type, gender and even race (Table 2B).

### Influence of Age, comparison with previous results

**Figure 1:**
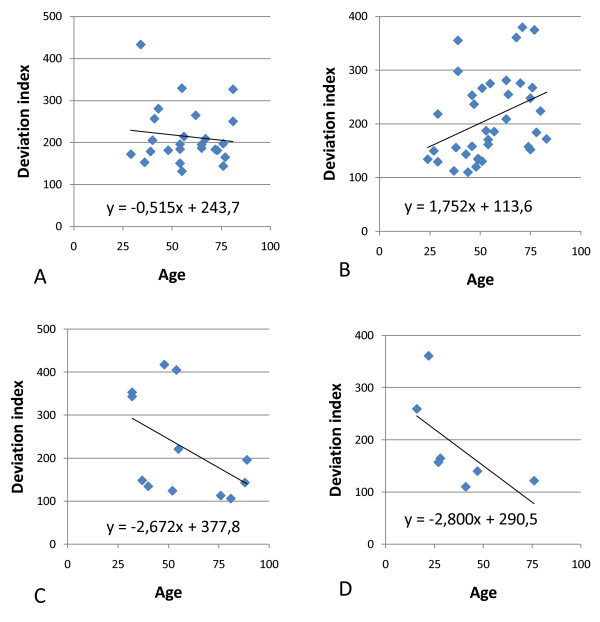
**Relationship between age and deviation index for A) females of M libraries, B) males of M libraries, C) females of V libraries and D) males of V libraries**.

An earlier paper [2] indicated that increase in age was correlated with a decrease in deviations from the mean transcription profile. These deviations were expressed in a deviation index i.e. the standard deviation of all the deviations in a given library. In this paper this deviation index was calculated with database BB (Additional file 2). Since Database BB only consists of deviations from the expected mean expression of 1000, calculation of the standard deviation of these deviations for a given library results in the deviation index for this library. To determine whether a similar age effect; as observed in the previous database; is seen with the new muscle database, deviation indices were calculated for all libraries in the four groups and were examined for any relationship to donor age. An ANCOVA for the M libraries showed that there is a gender-specific difference for the relationship between age and deviation index (P = 0,038). In the ANCOVA for the V libraries such a correlation was not observed (P = 0,960) although there was some indication of an age-effect (P = 0,081). To visualize these effects regressions relating age and deviation index for the four groups are shown in Figure 1. This figure suggests that the deviation index goes up with age for M males and goes down in the other three groups. Therefore the results obtained with the muscle database also suggest an influence of age on the deviation index that can be gender and tissue specific, thereby confirming previous findings obtained with a database comprised of a variety of normal tissues. In contrast with the previous paper also an increase in deviation index with increasing age was observed in one of the groups (M males) which is a new observation.

The previous paper [2] also showed that the age-specific alteration in deviation index occurred mainly in a subset of the probes. In order to investigate this further using the muscle database, the probes were sorted according to their contribution to this age effect. To obtain an estimate of this contribution the relationship between age and residual errors was established for each probe.

**Figure 2:**
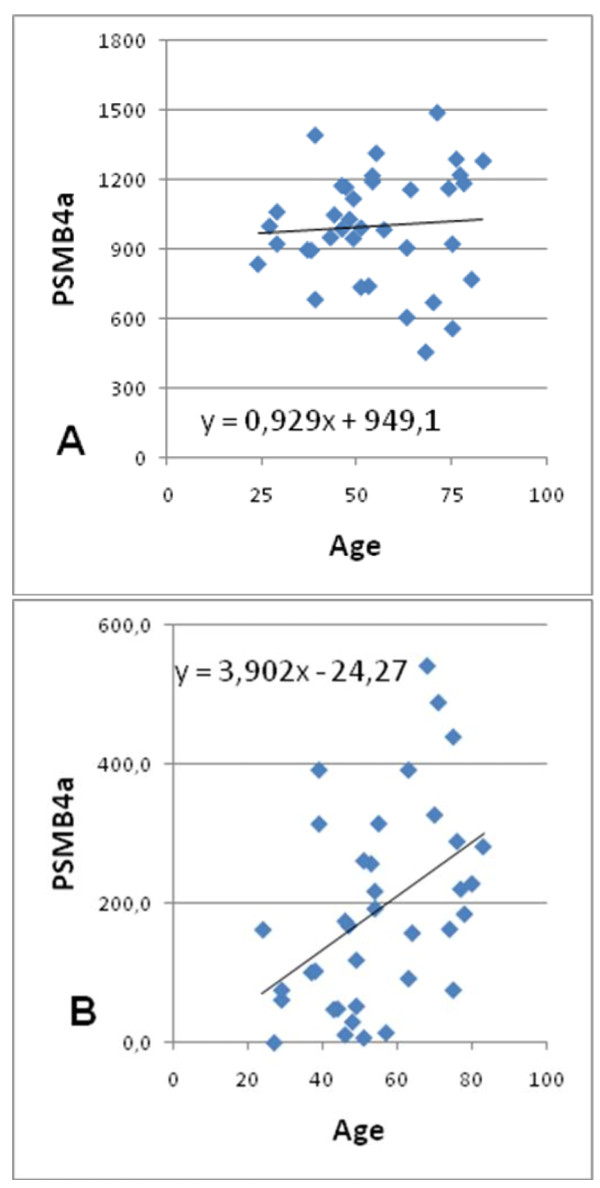
**Illustration of procedure used to evaluate the contribution of a probe to an effect of age on deviation index**. Figure 2A shows the transcript abundance of probe PSMB4a in database BB of males in M libraries To generate the residual errors in Figure 2B, all data points were reduced with 1000 and resulting negative residual errors were converted to positive values. In this case of PSMB4a the regression with age is positive and significant (P = 0,005).

The procedure, illustrated in Figure 2 for probe PSMB4a, is as follows: residual errors are obtained by subtracting 1000 from each data point in database BB. Half of the resulting residuals will be negative and are transformed to positive absolute values.

When the regression analysis between age and these absolute residual values for a specific probe is significant, then the probe in question has residual values which increase (or decrease) with age and will thus contribute to an age effect.

For M males, 18 of the 62 regression lines had a negative slope and 44 a positive slope, this difference from a 1 to 1 ratio being significant (P = 0,00096) indicating that for many slopes there is an increase of the residual value with age. Eight of the positive slopes were significant (P < 0,05). Using the same procedure for all data, the regression lines between age and the absolute residual values was determined for each of the four groups, 62 regression lines per group. The four groups differed in numbers of regression lines with a positive or negative slope. While M females did not show a significant deviation from the expected 1:1 ratio, V males and V females did (P = 9,61 × 10^-8 ^and P = 2,29 × 10^-8 ^respectively), both with an excess of negative slopes (Table 3).

**Table 3:** Age and deviation index.

	**rectus abdominis (M libraries)**				**muscles of extremities (V libraries)**			
	**females**		**males**		**females**		**males**	
**no of libraries**	25		37		12		7	
**slopes of regression of age with residual values**								
	**p-value**		**p-value**		**p-value**		**p-value**	
**negative slope**	36		18		52		53	
**positive slope**	26	0,20408	44	0,00096	10	9,6E-08	9	2,3E-08
**regression of age with deviation index of probeset**								
	**Slope**	**p-value**	**slope**	**p-value**	**slope**	**p-value**	**slope**	**p-value**
**all 62 probes**	-0,515	0,577	1,75	0,024	-2,67	0,120	-2,80	0,131
								
**first 5**	-4,36	**0,006**	-1,63	**0,035**	-2,20	**0,013**	-2,16	0,065
**first 10**	-3,56	**0,007**	-0,73	0,196	-1,91	0,055	-1,51	**0,002**
**first 15**	-2,82	**0,026**	-0,62	0,183	-2,00	0,074	-1,57	**0,039**
								
**last 5**	2,33	0,227	4,26	**0,029**	1,19	0,197	0,20	0,750
**last 10**	1,67	0,230	3,81	**0,008**	0,90	0,233	0,11	0,765
**last 15**	1,37	0,270	3,72	**0,011**	0,55	0,391	0,19	0,437

Influence of probe selection on the relation between age and deviation index. Tissue and gender specificity.

Subsequently for each of the four groups (males and females from the M and V libraries) the probes were ranked in slope order from the most negative slope to the most positive and deviation indices were then calculated for subsets of the probes and their regression with age determined. Table 3 shows that the decrease or increase in deviation index with age depends on the subsets of probes used.

This analysis therefore broadly confirms our previous findings of the effects of age, gender and tissue-type on the expression of the proteosamal genes.

### Further analysis of the residual variation in expression

**Figure 3:**
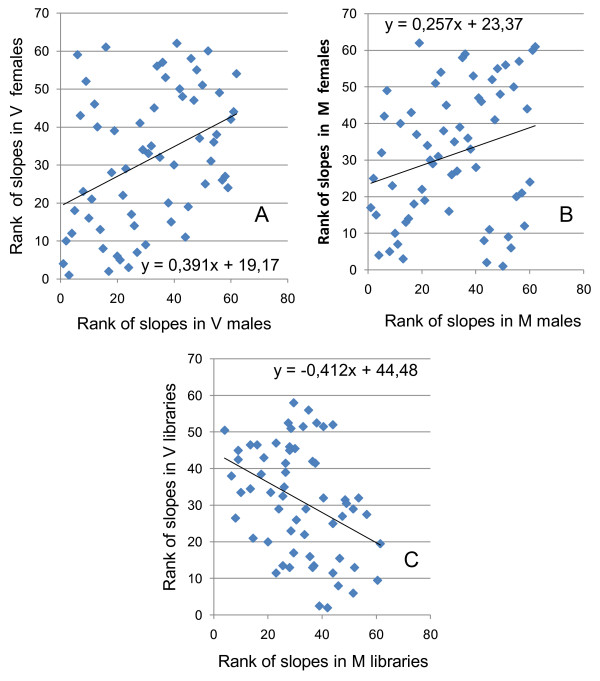
**Comparison of the slopes of the regression lines between age and the absolute residual values**. A) The slopes of males and females correlate in V libraries (P = 0,00166). B) The slopes of males and females correlate also in M libraries (P = 0,043). C) The slopes of M libraries and V libraries correlate also (P = 0,00164) but this correlation is negative !

Since this analysis shows that the probes differ in their contribution to the age-related change in deviation index, the question arises to what extent the same probes in all four groups are involved in these age effects. To answer this question, the slopes of the 62 regressions lines (regression of age with absolute residual values) of each group were compared with each other by ranking the slopes from 1 (lowest) to 62 (highest). The slopes of V females correlate with those of V males (Figure 3A, P = 0,0017), indicating similarity in the age effects of the probes. Similarly, the slopes of M females also correlate with those of M males (Figure 3B, P = 0,043). Therefore the probes that contribute to the age effect in the V and M libraries are similar in both sexes. To determine whether there is also similarity in probe contribution between M and V libraries, the mean ranks of females and males were calculated for both M and V libraries and then compared with each other. A strong correlation was found (Figure 3C, P = 0,0016), indicating that similar probes are involved in the age effects. However this correlation is negative (-0,39) meaning that the probes that show a reduction in residual value with age in the M libraries have an increase in residual value with age in the V libraries and *vice versa*. Therefore the epigenetic aging phenomenon appears different for the two different tissues although similar probes are involved.

### Prospects for further analysis: network of interactions

At present, although the association with age is clear, the cause of these age-related phenomena remains a mystery. However, this database lends itself to further analysis that may help clarify what is actually taking place.

Correlation matrices of expression files reveal the presence of intricate networks of probe expressions. In the M males BB database, about 32% of the 1891 correlations are significant (P < 0,01) with positive and negative correlations occurring in around equal numbers. The distribution of significant correlations between the probes is however not random. While some probes (A6, D05a and D05c) have no or only one 1 significant correlation, other probes like A7a, B4a, B6 and D04c are strongly involved with more than 35 of the 61 correlations being significant. This means that amongst the probes, subgroups can be distinguished that are characterized by a high positive correlation within the groups and a high negative correlation between the groups, Thus when a subgroup shows high expression, the second subgroup shows low expression and *vice versa*. This suggests that age-related oscillations in gene expression could take place. Further analysis might help to elucidate the nature of such oscillations.

### Summary of results

1. The transcription profile of the proteasomal genes is different for the M and V libraries both in degree and pattern of expression. Therefore the abdominal muscle can be distinguished from the skeletal muscles of the extremities on the basis of proteasome expression.

2. About 50% of the variation in transcript abundance is due to differences in proteasome expression level.

3. Since experimental error contributes only 1,4%, the remaining variation (about 50%) is due to inherent differences between the two tissue types and to constitutional factors (e.g. gender and race).

4. After removal of the effect of proteasome expression level on transcript abundance a gender and tissue-specific relationship was found between age and the deviation index (with both increase and decrease being observed).

5. The probes differ considerably in their contribution to the age effect.

6. Although in the four groups similarity exists in the degree each probe contributes to the age effect, the direction of their age effects is antagonistic for M and V libraries.

7. Interactions exist in the expression of the probes, resulting in a complex network

## Discussion

### Validation of previous results

A previous study, using a large number of normal tissues [2], indicated that the pattern of transcription of the 20S proteasomal genes depends to some extent on age, gender and tissue type. This finding has now been corroborated by the present study that uses only two types of muscle. Consequently, since there does not appear to be any indication that these influences relate to proteasome structure and function, it suggests that the influence of age, gender and tissue type on gene expression, without a concomitant relation to the specific function of the gene, is a widespread phenomenon and not just a quality of the proteasomal genes.

A possible explanation for this phenomenon is that it serves to maintain the differentiated state of the cell and is due to epigenetic factors that are gender and tissue-type specific and that are subject to age-related change. The need for such epigenetic factors is thought to arise because cells are complex systems (see discussion in previous paper [2]) with the proteasomal genes just reflecting this complex system as a "pars pro toto". The underlying structural basis in this complex system remains an enigma. The nuclear architecture could be one of the main factors involved.

The present study expands upon the previous findings. Whereas previously only a decrease in deviation index with age was observed, the present study shows an increase in deviation index with age for the males in M libraries. These age-related alterations are connected with a new phenomenon, i.e. with age there is a decrease or increase in residual variation between individuals in expression of the probes The degree of alteration in residual value is probe specific and the direction of the alteration (decrease or increase) is antagonistic in abdominal muscle against skeletal muscles. The reason for this age- related change remains, thus far, elusive, but is felt that elucidation of the phenomenon will be important for understanding the aging process.

Other new findings relate to the differences between tissues, to the presence of an intricate network of gene expressions, to the quantification of factors involved in the variability in expression and to improvements in the method of analysis.

### Relation to some other studies on gene expression variation with age

Variation in gene expression with age has been the subject of study on several occasions suggesting increase in variation in aging tissues [6-8] although this increase might be restricted to non renewing tissue [9]. Our foregoing study suggested that aging goes with a decrease in variability between individuals [10] while the present study suggests that both decrease and increase in variation between individuals can occur depending on the tissue and probe used. It is too early to judge this discrepancy as conflicting data since our approach is totally different. Our approach studies the pattern of gene expression in a group of functionally related genes and quantifies the degree of deviation from this pattern in a cohort of individuals of different age. Care was taken to remove differences in expression level from the data by correction for differences in proteasome expression level and by transformation of the data for removal of the differences in degree of expression of the individual probes. Our analysis therefore deals with the age related change in a pattern of expressing genes and not to an increase in noise or destabilization. Whether the observed changes lead to stabilization or destabilization is still an open question and the same holds for the involvement of stochastic events.

### EPIGENE method

Progress has been made in developing a method for analysing epigenetic hereditary profiles in gene transcription. Whereas previously the method consisted of establishing an index for all deviations from the mean transcription profile, the present method consists of the following steps:

1) Establishing more homogeneous subgroups in the database, e.g. for gender and tissue type.

2) Establishing the effect of factors that contribute to the variability in each library. Presently these factors are proteasome expression level and experimental error. The contribution of proteasome expression level to the variability can be removed and in this way a database is generated in which the individual libraries have the same mean proteasome expression level.

3) After setting the mean expression of each probe to 1000, a database with residual variability results that possibly only reflects constitutional differences between individuals that are probably mainly epigenetic in origin. In the case of the proteasome, this variability is still about 50% of the original variability (Table 2).

4) Analysis of the residual variability. Presently only one characteristic has emerged: increase or decrease of the residual variability with age. Since a complicated network of positive and negative correlations between the residual values of the probes is present and since the nuclear structure could be involved, further steps in the EPIGENE analysis appear possible.

## Materials and methods

The database of human muscle from GEO, GSE5086 was used [11]. This database consists of 81 libraries, 62 derived from the rectus abdominis and 19 taken from skeletal muscles of the extremities. The donors, 37 females and 44 males, varied in age from 16-89 years.

The transcription profile of the genes that code for the 26S proteasome was chosen for analysis. In the database 62 probes are available to study the expression of 33 genes:

1 probe for PSMA4, A6, B3, B6, C1, C3, C4, C5, C6, D2, D3, D7, D8, D14,

2 probes for A2, A5, A7, C2, D1, D6, D9, D10, D11, D12, D13,

3 probes for A1, A3, B1, B2, B4, D4, D5 and

5 probes for B7.

For PSMB5 no probe is present.

When more than one probe is available for a particular gene, the gene symbol was extended with alphabetic characters. The probe sets and their expression data are given in Additional file 1. All calculations were performed with XLSTAT

## Competing interests

The author declares that he has no competing interests.

## Reviewer's comments

### Reviewer's report 1

Dr. Thiago M. Venancio (nominated by Dr. Aravind Iyer), NCBI-NIH, Bethesda, Maryland, United States

#### Reviewer comments on the final version of the manuscript

After reading the revised version of the manuscript and the response to my comments, I still think that several assumptions of this study are likely to be wrong and the data sampling and methods are insufficient to support the author's conclusions. These two factors could possibly undermine the obtained results. Moreover, the main points discussed in my previous report were not satisfactorily addressed, as they required additional and deeper analyses and not just modifications in the text. Therefore, I think this manuscript does not meet the scientific standards of *Biology Direct*.

#### Reviewer comments on the original version of the manuscript

##### Major points

In the present paper, Simons addressed the interesting and complex problem of epigenetic effects on transcription, aiming to find gender-, tissue- and age-specific variations. To achieve this goal, he presented a novel method to measure the impact of the different factors to the gene expression variability.

First and foremost, although I consider epigenetic control on gene regulation an important topic, I do have considerable criticisms on this manuscript, regarding the fundamental assumptions, the way the problem is addressed and the drawn conclusions. I detail my observations below, along with suggestions to improve the manuscript.

The *Background *section is insufficiently detailed to give the reader a good introduction to the topics covered by the manuscript. This section has to be improved, with more references to the previous works, instead of merely citing topics followed by *etcetera*. It was surprising to see how a paper covering such an extensively studied topic can have only seven references (including one link and two self-citations).

Author response

*This paper does not cover an extensively studied topic since it deals with a new phenomenon. It would have been easy to include many references on well-known epigenetic factors such as DNA methylation, nuclear architecture, chromatin structure, histone modification, genomic imprinting, regulatory elements etcetera, but in my opinion that would not have resulted in an improvement of the "background". Instead the paucity of references underlines that a phenomenon is under study that so far was not. Unfortunately this might give the impression of disregard of published data. Therefore in order to avoid such misunderstanding some small changes in the text were provided*.

The author claims in the *Background *section that the proteasome *does not play a role in gene regulation*, which is not true for obvious reasons. The proteasome exerts extensive control over several different classes of proteins and their underlying biological processes. Therefore, it certainly encompasses (epigenetically and non-epigenetically) gene regulation and transcription [12-14]. I am not sure how critical is this assumption for the method, but it potentially undermines the model. In addition, some proteasome subunits are differentially used upon specific cellular signals. If some of these subunits were probed in this study, an additional source of noise might be expected.

Author response

*This role of the proteasome was new to me. Thanks. Therefore this sentence was removed and in the section on heterogeneity caused by proteasome expression level this possible source of additional heterogeneity has been mentioned*.

The methods section of the article is extremely poor. Although the author describes the methodology in the *Results and discussion *section, the text is inaccurate and hence it would be virtually impossible to reproduce the obtained results. In particular, I am still uncertain about what the author calls as *microarray database *and *libraries*. The datasets are not properly cited and the data processing to generate the tables is not described. The term database is also used in different contexts across the text. Is this a relational database? Is this a dataset? Is this a database of one dataset? What is the accession numbers of the dataset(s)? How the microarray data were processed? Which platform(s) was (were) used? All these questions must be explicitly answered for an adequate understanding of the study.

Author response

*Since the whole paper is in essence a search for a method to study epigenetic variation in gene expression the most of the section of "Results and Discussion" is on methodology and it has been the intention to deliver the data in such a way that results can be reproduced. However your remarks are to the point as to my dismay a small but essential section on "Materials and Methods" appeared to be missing from the text. This section has been re-inserted. My apologies for this shortcoming*.

An additional column with a unique identifier (e.g. Gene ID) could be provided in the table 1. This would help the reader to make an unequivocal reference to the gene in the public databases.

Author response

*Gene id's have been added to table *1.

In terms of biological results, I think the dataset is definitely too small and unbalanced to draw the conclusions presented in the paper. Even if these results are corroborated with an adequate number of samples, general biological inferences cannot be done with such small number of (related) genes. There are also technical concerns, as microarray and SAGE have their inherent limitations. It is not uncommon to reach discordant conclusions when comparing the results obtained by the two techniques. In addition, noise in gene expression was recently shown to be widespread at the cellular level. If one considers the difference between individuals, with different ages, gender and life-styles, the number of variables affecting gene expression is enormous and does not allow such direct conclusions. Therefore, I think the conclusions are unsupported by the presented data.

Author response

*Strictly speaking the results with these dataset, even although they corroborated earlier results with another dataset, hold only for the proteasomal genes. However since no discordant observations were made, the possibility of a general biological significance cannot be denied when the number of genes is small The degree of noise in gene expression is not a sett led item and in fact our results indicate that effects of age and gender should be distinguished from noise*.

As pointed above, I do not have the required details for a deeper evaluation of the method. However, I suggest some simple analyses. 1) Principal (or independent) component analysis would aid to recover important variables (components) affecting gene expression; 2) The statistical significance of the findings could be evaluated through a comparison of the obtained results with randomized datasets.

Author response

*1)) The Principal Component Analysis was applied in the previous paper and identified an effect of age and proteasome expression level on the variability in expression, which led to a further analysis. The present paper investigates whether the results of that analysis could be corroborated using another dataset. Since this appeared to be the case, a PCA was not opportune.2)) The use of a randomized dataset could certainly be of some value to exclude inappropriate handling of data. However one can already beforehand exclude the possibility that 2 libraries (from different donors) are so alike each other in gene expression that the experimental error (noise) can be at the most 3,5%. In fact this finding indicates that most of the variation in gene expression is due to variables and not to noise*.

Regarding the methodological contribution of the manuscript, it lacks several desired/required characteristics of a methods paper, such as statistical formalism and background information, benchmarking with other methods and simulations with randomized datasets.

Author response

*For me it is understandable that this paper leads to many more questions than answers and probably one can wonder whether the approach is the best one. However presently this is as far the method has been developed and these remarks cannot be translated by me into further improvements*.

In addition, open source programs/libraries are highly appreciated by the scientific community, in order to give free access to the heart of the scientific discoveries and permit a fair comparison with other techniques. In my opinion, free (of free speech) software should be mandatory for methods papers

Author response

*The missing reference that gives free access to the libraries of muscle has been included and XLSTAT is a free software*.

'In the present format, I think this paper is below the scientific standards and therefore do not support its publication in *Biology Direct *with the present format. I may recommend the acceptance as a *hypothesis paper *after re-evaluation of the extensive modifications'

##### Minor corrections

- The numbers are not formatted according to the journal recommendations. Comma is used instead of decimal delimiters.

- The quality/resolution of the figures is very bad. The ink-to-data ratio and the histogram color combination are also inadequate.

- There is a reference to a *figure z*.

- There are two *References *sections (one is empty).

- Blank pages in the PDF file.

- Additional files could be provided in plain text format to allow a better access by other scientists.

- The Geneatlas link is broken.

- There is an *Addendum *in the paper. This part could be included in the main text.

I declare that I have no competing interests.

Author response

*In the final version care will be taken that the paper and the additional files meet the official requirements if that would not be the case. Figure z has been changed into figure 5. One" Reference" heading has been removed. The link to Geneatlas was restored. The addendum became superfluous*.

### Reviewer's report 2

Dr. Hua Li (nominated by Arcady Mushegian) and Dr. Arcady Mushegian, Sowers Institute, Kansas City, United States

Most of the statistical models and analyses throughout the paper should be discussed in more detail.

Specifically,

1. Page 5 par 2: "To make the expression of the probes comparable, the distributions of each probe is transformed such that the sum of the expressions for each probe amounts to 100." Could you explain how this was done exactly, and why were the data rescaled to 100?

Author response

*The data were rescaled in order to make the expressions of the probes comparable and to to determine in a specific library the number of probes with exceptionally high or low expression (actually the transformation was to a distribution with a mean of 0 and a standard deviation of 1, this has been corrected in the text*.

2. Page 5 par 3: Which factors were included in ANOVA model? Why only 19 M libraries were used rather than all M libraries? Ideally, one could fit a model including gene, muscle type, gender, age and their interactions and decide if there is significant effect for any of these factors.

Author response

*Only 19 M libraries were used since only 19 V libraries are available and the ANOVA required equal numbers of libraries in the two groups. The subsequent ANCOVA that has not this requirement suggested an interaction between gender and age*.

3. Page 6: the author shows that there is no age effect for gene expression. If this is the case, then comparisons between slopes generated for males and females do not make any sense because all slope estimates are essentially zero. Also, why Wilcoxon rank test was used here rather than a t-test?

Author response

*In the revision the part on the effect of age on transcript abundance in males and females was left out as it was of secondary importance*.

4. Page 6, bottom: The "proteasome expression level" for each library is the sum of expression levels of all genes in that library, correct? What is the goal of regressing the proteasome expression level on the expression of individual probes?

Author response

*The individual probes do not contribute to the same extent to the total proteasome expression level in the different libraries. As discussed in the previous paper this might be connected with the observation that some of the probes identify transcripts that result from constitutive expression while other probes identify transcripts that could have a rate limiting function. By regressing the proteasome expression level on the expression of individual probes the variation caused by this phenomenon can be ascertained and removed from the dataset*.

5. Page 7: How were the variances and the degrees of freedom of libraries M19 and M25 (page 7) calculated? A more detailed description of the model is needed. For any statistical analysis, the assumption is the error has a normal distribution with mean 0 and certain variance. Since M19 and M25 are two samples from the same types of muscle, the error variances should be similar - why the conclusion that the errors are due to epigenetic factors?

Author response

For each probe the variance was calculated with the two expressions in the two libraries. With these 55 variances of the 55 probes, each with one degree of freedom, the overall variance with 54 degrees of freedom was calculated. Presumably, since this variance is so small, it could reflect the experimental error or noise. When the experimental error is so small, the remaining variability must have a biomedical meaning. It is indicated that a large fraction of the variability is due to constitutional factors like gender, tissue type, age and race. The influence of these constitutional factors suggests that they cause fixed programs in the transcription of the genes. Here It is supposed that this reflects the epigenetic programming known from animal cloning

6. Page 10, par 1 and 2: P-values < 0.01 are mentioned twice, in connection with "significant probes". What was the hypothesis, e.g., was it whether the correlation coefficient (r) was different from 0, or from a different number, or something else? If the former is the case, could one conclude that correlation is significant just because "r" is not zero?

Author response

*I am not sure whether I understand your question correctly. In the first paragraph it was investigated whether specific probes are more involved in the differences between the two types of muscle or in the differences between males and females. Not a correlation coefficient was used but the F ratio: the ratio of the variance in expression of each probe e.g. "variance A1a males"/"variance A1a females" etc. Significant F ratio's were observed for a fraction of the probes only, indicating specific involvement of some of the probes. In the second paragraph a correlation matrix was determined to compare the expressions of each probe in the different libraries. Significance indicates that r is different from 0*.

7. Page 10, "Prospects for further analysis": what is "networks of expressions" - what are nodes and edges in such a network? Are nodes probes, and edges strong correlations of expression levels, as the paragraph seems to imply? If so, how was the P-value computed?

Author response

*A Principal Component Analysis, performed with XLSTAT, provides also a correlation matrix with the correlations between the expressions of the individual probes and indicates at the same time whether the P-values of these correlations are significant. The abundance of significant correlations indicates a network: a strong interrelation of the residual variation in expression (presumably epigenetic in origin). The source of this interrelation is not known but could be an epigenetic structure e.g. the structure of the nucleus*.

8. Page 11, Summary, #1: what is the difference between degree and pattern? #9: change "nucleus" to "genome".

Author response

With degree is meant that the level of expression of the probes is higher in M libraries than in V libraries. With pattern is meant that the ratio of expressions in M an V libraries is not fixed but different for different probe; "nucleus" has been changed into: "genes and transcripts"

### Reviewer's report 3

Dr.J.P.de Magelhaes (nominated by Dr.G. Church), School of Biological Sciences, University of Liverpool, UK

#### Reviewer comments on the final version of the manuscript

I think the manuscript is better now and the author successfully addressed some of my concerns. The analyses are better justified in the revised ms and the discussion is improved. However, a few problems persist. In particular, I am still concerned about the significance of some of the statistical analyses. For example, the author still uses p = 0.05 as threshold for testing the statistical significance of the slopes of the probes (page 7), which indicates that no correction was done for multiple hypotheses testing. Lastly, I maintain my opinion that, even though gene expression variation with age is an interesting subject, this work only slightly advances our understanding of it.

#### Reviewer comments on the original version of the manuscript

In this work, the author employs data from human muscle to study gene expression variation in genes encoding the 26S proteasome. While in some tissues gene expression variation appears to decrease with age, in others it increases (also depending on gender). The author attributes these differences to specific genes. I thought the topic was quite interesting and timely, yet unfortunately I found very little new or surprising insights in the results. Moreover, the ms has numerous problems, as described below.

Although I think the idea that gene expression variation changes with age intriguing, this has been reported previously by others, including the author. The author mentions that the increase in the deviation index with age for males in one of the tissues is novel, but as detailed below I do not think this is statistically significant. So the results seem to me to be mostly confirmatory of earlier findings. Even if the results are significant, it is not clear to me what they mean as the author reports that the deviation index increases with age in some tissues (or gender) but not in others. The author does not appear to offer any explanation for this discrepancy either, so the relevance of these results eludes me.

The last paragraph of the discussion mentions the new findings reported in the ms, one of which was "the possible involvement of nuclear factors" which I could not understand how it relates to this study since no nuclear factors were studied. The author also reports "a complicated network of positive and negative correlations" between the probes, but I was not surprised by this. I would expect an analysis focusing on a given protein complex to find correlations between the expression profiles of its individual components.

Author response

*The paragraph mentioning the "correlation network" and the "possible involvement of nuclear factors" had no other intention than to explore further possibilities for research aimed at clarification of the new phenomenon. The finding of negative correlations between frequencies of individual components of the proteasome is puzzling and not to be expected. Since the part on possible involvement of nuclear factors is not essential for understanding the ms, this part of the paragraph has been removed*.

One major concern I have with this work is the statistical analyses, for which very few details are given. Importantly, I am not convinced that many of the results reported are statistically significant since given the multiple hypotheses tested some correction is necessary. A Bonferroni correction, for instance, would render the results of figure 1 no longer statistically significant with p = 0.024. For testing the statistical significance of the slopes of the probes the author uses p = 0.05 as threshold, which again does not take into account multiple hypothesis testing. The way the slopes of the regression lines correlate between males and females appears to be significant and the negative correlation in the M libraries was intriguing, though I am not sure what the latter means biologically.

Author response

*As stated in the ms an ANCOVA on the M libraries indicated a significant interaction of age and gender on the deviation index (P = 0,038)confirming previous results. Further analysis is based on this finding and *figure 1*only serves to visualize this result and as you remarked the p-values of the 4 regression lines in *figure 1*do not give much information. Therefore these p-values have been removed from the ms and an additional sentence was added to the text. That the result is highly significant follows from the further analysis given in table *3* and *figure 3.

A number of analyses seem to be arbitrary with no justification. For example, the author calculated an "approximation of the experimental error" using the correlation coefficient of two samples, but I found no explanation for how these two samples were selected.

Author response

*Within the group of M females the mean and standard deviation of the correlation coefficient is -0,035 and 0,477, therefore some libraries will be rather similar. The libraries M19 and M25 have 0,985 as correlation coefficient and have a highly significant regression (P = 2,31 × 10^-46^, after Bonferroni correction P = 7,49 × 10^-43^). This means that these two libraries are very much alike in expression and therefore could approach the similarity of repeats. This explanation has been added to the text*.

A few other minor comments:

A central thesis of the work is that this variation in gene expression has an epigenetic basis. While this indeed might be the case, I found no direct evidence supporting these claims. I can conjure other explanations for gene expression variation, such as stochastic variations in transcription factor levels between cells that are augmented with age, DNA damage accumulation that is random by nature, etc. I would suggest that the author considers alternative explanations for the results.

Author response

*So far there is no proof that this variation in gene expression is epigenetic. This would be indicated when the variation is hereditary for which is some evidence: 1) similarity in deviating patterns in gene expression between a tumor and the normal tissue from which it was derived or between a tumor and its metastasis (paper I), 2)race related variation in expression (this paper), 3)specific expression patterns for the two tissues suggest that the patterns are hereditary, 4)the decrease or increase in deviation index with age does not support random stochastic events as driving force. As suggested alternative explanations could be possible but the phenomenon seems at present too new and unknown to make a more meaningful discussion possible*.

As mentioned by the author, some probes had very low levels. Can probes with such a low signal intensity be classified as being expressed (i.e., above background)?

Author response

*Interesting question. When I take the libraries M19 and M25 as repeats, the expressions of the probes range from 19,5 to 4387. From each pair of signals the mean and standard deviation was calculated to obtain a noise to signal ratio. All the obtained standard deviations are around 2,6 percent of the mean and also do not show a tendency to approach the mean value at the lowest expressions (1,0% at expressions >1000, 2,9% for expressions ranging from 250 to 1000, 3,9% for the range of 100 to 250 and 4,4% for the range of 20 to 100). Therefore the noise in all the determinations appears to be negligible small and all signals appear well above background*.

Lastly, there is no discussion of how the results reported in the ms fit with other similar works. Specifically, other studies have shown increase in gene expression variation with age:







There is also evidence that increased transcriptional instability with age may be more significant in nonrenewing tissues:



Author response

*This is of course a question. To explain that there is as yet no fit with other studies and why, the discussion has been improved by adding an additional paragraph*.

In conclusion, while the ms has some potentially new results, it is mostly confirmatory of previous findings. I also think the statistical analysis has errors and lacks clarity. In my opinion, the weaknesses of the manuscript in its present form outweigh its strengths.

*Author response to*:

"1)very little new or surprising insights in the results, 2) Although I think the idea that gene expression variation changes with age intriguing, this has been reported previously by others, including the author, 3) So the results seem to me to be mostly confirmatory of earlier findings. Even if the results are significant, it is not clear to me what they mean, 4) the relevance of these results eludes me."

*Indeed, the relevance of the findings is still a mystery and that will be the case as long as there will not be many more data available. It seems still too early for a fully grown hypothesis. However my thinking to explain the findings is developing into this direction: First, the variation studied is not variation in terms of noise. Quite the opposite seems to be the case: the noise is very low and very stable patterns of proteasome expression seem to be present but with a large variation in these stable patterns between individuals and between tissues. Changes have to occur in these patterns during development since the tissues (which have different patterns of expression) arise from one fertilized egg (which is bound to have its own pattern). Secondly let's assume that gene expression is always partly due to an epigenetic structure and that this structure is transmitted to daughter cells. Although in this way the pattern of proteasome gene expression will be preserved during cell division small changes will occur. The type of tissue and also gender and age appears to influence the direction of these epigenetic alterations. An additional assumption could be that the epigenetic changes are unidirectional: young tissues might have a less developed epigenetic structure than old tissues. Gene expression in old age could than go with a finer tuned epigenetic expression profiles (and decrease in deviation index) but with a loss in flexibility in expression*.

*These ideas can develop into a completely new hypothesis on ageing, a theory that is not based on accumulation of damage to macromolecules and also not based on a regulatory programme. The new method developed here will allow the testing of predictions made on the base of this hypothesis. The use of a pattern of expression and a deviation index from this pattern for a group of functionally related genes is new and the presence of age associated alterations in this index observed previously had to be confirmed in my opinion with another database (indeed confirmatory)*.

## Supplementary Material

Additional file 1 **Expression of the proteasomal genes**. Database A: raw data; Database AA: expression of each probe transformed to a mean of 1000. Click here for file

Additional file 2 **Expression corrected for proteasome expression level**. Database B: correction of database A; Database BB: expression of each probe in database B transformed to a mean of 1000. Click here for file
